# A colorimetric detection of Hg^2+^ based on gold nanoparticles synthesized oxidized N-methylpyrrolidone as a reducing agent

**DOI:** 10.1038/s41598-023-49551-x

**Published:** 2023-12-14

**Authors:** Xiaodong Shao, Dou Yang, Min Wang, Qiaoli Yue

**Affiliations:** 1 State Key Laboratory of Performance and Structural Safety for Petroleum Tubular Goods and Equipment Materials, CNPC Tubular Goods Research Institute, Xi’an, 710077 China; 2 School of Chemistry and Chemical Engineering, Northwestern Polytechnical University, Xi’an, 710129 China; 3 School of Chemistry and Chemical Engineering, Shandong Provincial Key Laboratory of Chemical Energy Storage and Novel Cell Technology, Liaocheng University, Liaocheng, 252059 China

**Keywords:** Chemistry, Nanoscience and technology

## Abstract

In this study, a gold nanoparticles colorimetric probe (AuNPs) with direct response to mercury ions (Hg^2+^) were developed using treated N-methylpyrrolidone (NMP) and chloroauric acid (HAuCl_4_) as precursors. NMP showed good reducibility after high temperature hydrolysis and could be used as reducing and stabilizing agent to synthesize AuNPs. The prepared AuNPs have obvious characteristic absorption peaks and appear wine-red. At the same time, it was found that the presence of Hg^2+^ can cause the aggregation of AuNPs, increased the absorbance at 700 nm, and changed the color of the solution into blue-gray. This method is capable of sensitive and specific determination of Hg^2+^ ranging from 1 to 30 μM, with the limit of detection (LOD) at 0.3 μM. The method showed good specificity for the determination of Hg^2+^ and has the potential to be applied to Hg^2+^ detection in sewage samples in the environment.

## Introduction

Heavy metals not only pollute the environment, but also have a negative impact on human health. Mercury (Hg) is a heavy metal element naturally present in air, water and soil. It can be widely distributed in the environment through natural and human activities^1–3^. Hg^2+^ exists in both inorganic and organic forms, which belongs to persistent toxic pollutants and can be enriched in organisms through the action of the food chain^4^. Causes a range of health problems such as myocardial infarction, Minamata disease, and some autisms by damaging the kidneys, central nervous system, and reproductive system^5,6^. Traditional methods for detection of Hg^2+^ often require expensive, complex and bulky instruments, and the operation process is complicated, which makes it difficult to meet the needs of routine analysis and field analysis^7–9^. Such as atomic absorption spectrometry, mass spectrometry and high-performance liquid chromatography^10–12^. Therefore, there is an urgent need to explore a highly sensitive, selective, and easy to operate Hg^2+^ detection method to detect Hg^2+^ in the food chain and environment, in order to protect the global food and water environment from pollution and prevent harm to human health.

Recently, many sensing probes for Hg^2+^ detection based on colorimetric detection and fluorescence turn on sensing are being developed^13–15^. At the same time, the advantages of gold nanoparticles (AuNPs), such as good biocompatibility, easy chemical functionalization, and unique optical properties, have been gradually understood^16–21^. In the Ultraviolet–visible (UV–vis) absorption spectrum, the position of the absorption peak produced by AuNPs is related to its particle size (or aggregated state) and shape, and the intensity of the absorption peak is linearly related to the concentration of AuNPs in the solution^22,23^. Therefore, AuNPs has unique advantages in the development of colorimetric sensors. For example, AuNPs-based colorimetry has been widely used to detect Hg^2+^. Recently, Ma et al. successfully constructed a novel label-free colorimetric sensor, and for the first time proposed Co^2+^ as exonuclease III cofactors. Using auxiliary signal amplification and unmodified AuNPs as indicators to achieve Hg^2+^ ultrasensitive detection^24^. Gosavi’s group reported that the successful used of AuNPs /rhodamine B (RB)/hexanedithiol (HDT) nanocomposite system for highly selective detection of Hg^2+^ in urine and ground water^25^. Chen et al. proposed an amalgam formation method using gold, combined with Hg^2+^ mediated the growth of AuNPs, which colorimetric sensing assay capable of efficient and rapid detection^26^ of Hg^2+^. However, in these methods, some ligands contain sulfhydryl groups and have a strong sulfide odor, and the detection of Hg^2+^ are based on multi-step or complex systems, and the detection process is complicated. Therefore, an environmentally friendly colorimetric probe that can directly respond to Hg^2+^ is urgently needed.

In this work, using pretreated N-methyl pyrrolidone (NMP) as reducing and stabilizing agent, an AuNPs colorimetric probe with direct response to Hg^2+^ was developed, avoiding the use of environmentally unfriendly thiol ligand-like ligands. The presence of Hg^2+^ can be caused the aggregation of AuNPs, the absorbance at 700 nm increased and the solution color changed from wine-red to blue-gray were observed, which is easy to identified with the naked eye. These results show that the method has good selectivity for the detection of Hg^2+^. The high sensitivity, specificity and convenience of Hg^2+^ detection have been achieved, which lays a foundation for further detection of Hg^2+^ in environmental sewage samples.

## Experimental section

### Chemicals

NMP was supplied by Tci Development Co., Ltd. (Shanghai, China). Including NaOH, Fe(NO_3_)_3_, CuCl_2_, HgCl_2_, Zn(CH_3_COO)_2_, Pb(NO_3_)_2_, MgCl_2_, CoCl_2_, BaCl_2_, CrCl_3_, NaCl, NiSO_4_·6H_2_O, CdCl_2_, CaCl_2_, HCl, HNO_3_ and chloroauric acid (HAuCl_4_) metal salts were acquired from Aladdin Reagent Co., Ltd. (Shanghai, China). All these chemicals are of analytical grade and used directly. Deionized water provided by the Milli-Q water purification system was used for the entire experiment.

### Apparatus

The UV-750 spectrophotometer (PerkinElmer, USA) was used for measurement of UV–vis absorption spectra. The morphology and size of AuNPs were recorded on a Talos F200X transmission electron microscope (TEM, Thermo Scientific Ltd., USA). The TEM used a common copper grid to load the sample and operated at an accelerating voltage of 200 kV. Fourier Transform infrared (FT-IR) spectra were carried on a Nicolet 6700 (Thermo Scientific Ltd., USA), used the KBr method. X-ray photoelectron spectroscopy (XPS) was performed on a K-Alpha spectrometer (Thermo Scientific Ltd., USA).

### Preparation of NMP*

The method of preprocessing NMP has been modified according to previous literature^27^. 50 mL of NMP and 50 mg NaOH were put in a 150 mL one-neck flask, and then refluxed at 160 °C for 12 h under argon protection. After the reaction was completed, a brown-yellow transparent solution was obtained. Centrifuged at 12,000 rpm for 30 min, and the supernatant was removed to acquired a pasty precipitation (NMP*). Dissolve the NMP* in 6.5 mL of deionized water and stored at 4 °C for use in the next step.

### Preparation of AuNPs

The preparation steps of AuNPs refer to the previous literature^28^. All reactions were performed in glassware thoroughly cleaned using aqua regia. 2400 μL NMP*, 800 μL ultrapure water and 100 μL of HAuCl_4_ (24.28 mM) were blended and stirred at 70 °C for 45 min. During this period, the color gradually changed from pale-yellow to wine-red and the pH gradually varied from 5.89 to 6.86, which proved that AuNPs were obtained. The cooled solution was stored at 4 °C for later studies.

### Colorimetric assay for the Hg^2+^

The Hg^2+^ detection procedure is described as follows: first, various concentrations of Hg^2+^ standard stock solutions were prepared by dissolving the metal salt HgCl_2_ in deionized water. Then the AuNPs system was mixed with various concentrations of Hg^2+^ at a 1:1 volume ratio and incubated for 10 min. The UV–vis absorption spectra were acquired at 700 nm in the presence and absence of Hg^2+^. The selectivity of AuNPs to Hg^2+^ was investigated by performing detection of other relevant metal ions.

### Pretreatment of real samples

In order to explore the practicability of the detection method, three environmental water samples were tested. The lake water was obtained from the artificial lake of Liaocheng University, the tap water was obtained from the chemical laboratory of Liaocheng University, and the river water was obtained from the local natural Tuhai River. After the water samples were precipitated for 24 h, the supernatant was appropriately diluted with deionized water. After adding different concentrations of Hg^2+^ standard solutions to the samples, the UV–Vis absorption spectra of AuNPs-Hg^2+^ at 700 nm were recorded.

### Ethical approval

The study did not involve human and/or animal studies, this statement does not apply.

## Results and discussion

### Principles of the colorimetric assay for Hg^2+^

**Figure 1:**
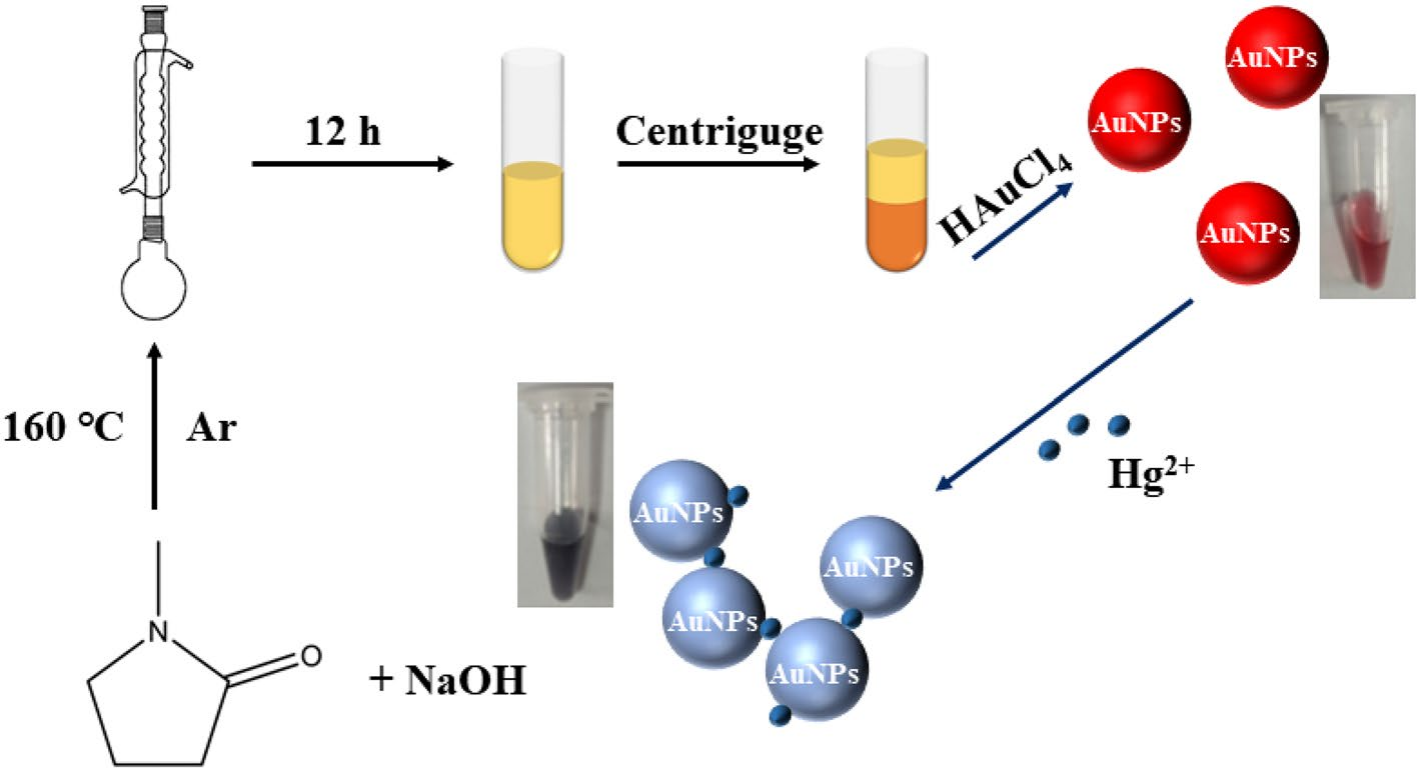
Schematic illustration for AuNPs synthesis and the detection of Hg^2+^.

In this study, developed a colorimetric assay for Hg^2+^ detection using AuNPs (Fig. 1). The AuNPs were prepared in the experimental section and displayed red color, according to previous literature^27^. NMP can be oxidized and hydrolyzed to formed NMP* with high reducibility under alkaline and high temperature conditions, and can be used as reducing and templating agents to prepared metal nanoclusters.

**Figure 2:**
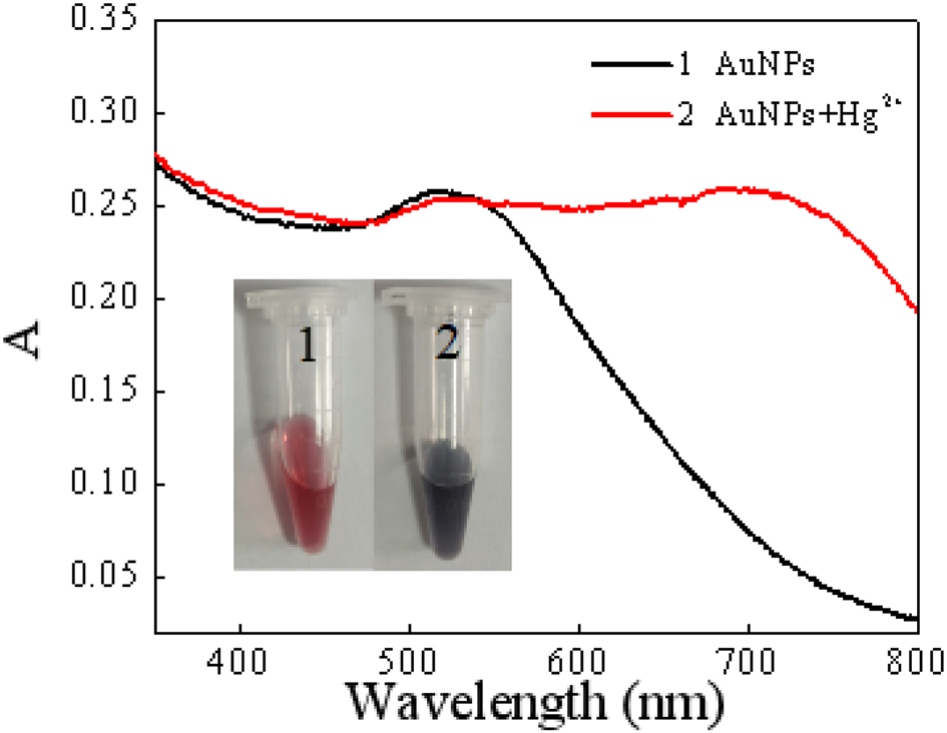
Absorption spectrum and photos of the AuNPs in the presence and in the absence of Hg^2+^.

**Figure 3:**
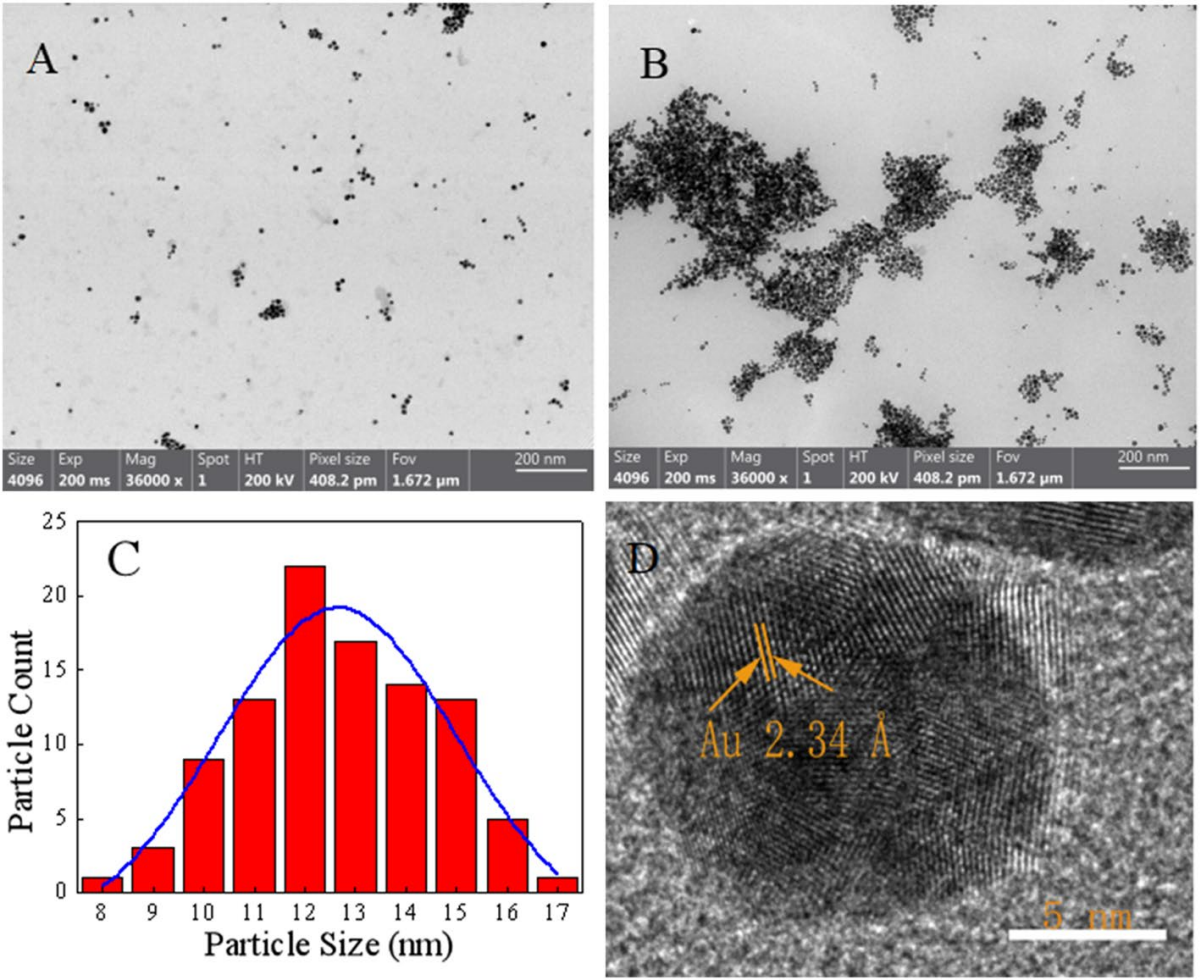
TEM images of AuNCs (**A**) and AuNCs + Hg^2+^ (**B**), particle size distribution diagram (**C**) of AuNCs, and HR-TEM images of AuNCs (**D**).

NMP* and HAuCl_4_ were used as precursors to prepared AuNPs with sensitive response to Hg^2+^. In the absence of Hg^2+^, a stable wine-red dispersion was observed and displayed a characteristic surface plasmon resonance (SPR) absorption band at 518 nm (Fig. 2). This is because AuNPs are anisotropic nanomaterials, and the absorption spectrum of AuNPs in monodisperse state has only a single peak at 518 nm^28^. The appearance of Hg^2+^ could induce the aggregation of AuNPs, so that the absorbance of AuNPs at 700 nm increased continuously, and the color of the mixture became blue-gray. This is due to the polarization and coupling between the electrons of adjacent nanoparticles when the AuNPs are in an aggregated state, resulting in a red-shift of the maximum absorption wavelength of the AuNPs. Correspondingly, the wine-red AuNPs gradually turned to blue-gray^29^. As shown in Fig. 3A,B, this aggregation process was also confirmed by TEM, AuNPs were in monodispersion without Hg^2+^. AuNPs were clearly aggregated together with Hg^2+^, resulting in a significant changed in color and providing colorimetric detection of Hg^2+^.

### Characterization of AuNPs

TEM, FT-IR and XPS were used to characterize the resulting functionalized AuNPs. The morphology of AuNPs was first investigated used TEM. The results showed that our as-prepared AuNPs were freely dispersed in water with good water solubility (Fig. 3A). As shown in Fig. 3C, by calculated, the particle size distribution of AuNPs was between 8–17 nm with an average diameter of 13 ± 2 nm. HR-TEM revealed the lattice fringes of 2.34 Å for AuNPs consistent with metallic gold (Fig. 3D), which corresponds to the d spacing of the (111) crystal plane of fcc Au^30^.

FT-IR spectroscopy was used to analyze the functional groups of AuNPs (Fig. S1A, in Electronic Supplementary Information). The broad band at 3430.86 cm^−1^ corresponds to stretching vibrations of inter molecularly bonded O–H group. The rest peaks observed at 2960.31 cm^−1^, 2890.61 cm^−1^ are attributed to stretching vibration of C–H, 1574.32 cm^−1^, 1650.00 cm^−1^ are attributed to stretching vibration of C=O, Absorption bands located at 1414.10 cm^−1^, 1410.51 cm^−1^ and 1300 cm^−1^ correspond to the C–N stretching vibration bands. The results indicate the successful synthesis of NMP* and AuNPs, as well as the oxidation of NMP into more reducing secondary alcohols^34,35^. Fig. S1B (in Electronic Supplementary Information) shows the HR-XPS spectra of AuNPs in the range of 82–90 eV. Au4f_7/2_ and Au4f_5/2_ are centered at binding energy values of 84.2 and 87.9 eV, respectively^31^. Au4f_7/2_ and Au4f_5/2_ could be further deconvoluted into two distinct peaks, respectively. The higher intensity doublets at binding energies at 84.2 (Au4f_7/2_) and 88.0 eV (Au4f_5/2_) could be attributed to Au^+^, while the binding energies of Au^0^ are lower at 88.3 (Au4f_7/2_) and 87.1 eV (Au4f_5/2_). According to previous literature, the difference between the peaks of Au4f_7/2_ and Au4f_5/2_ (about 3.7 eV) is caused by the presence of Au^0^, and in AuNPs, Au^0^ occurs in the metal cores protected by surface-capped ligands^32,33^ and Au^+^.

### Optimization of AuNPs synthesis conditions

The conditions were investigated by comparing the UV–Vis absorption spectra of AuNPs including the precursor ratio and temperature. Fig. S2A (in Electronic Supplementary Information) shows the UV–Vis absorption spectra of AuNPs prepared at volume ratios of NMP* and HAuCl_4_ from 3:1 to 8:1. When the ratio was between 6:1 and 8:1, the absorption spectra show characteristic absorption peaks. AuNPs prepared at 70 °C display the most obvious characteristic absorption peaks when the synthesis temperature was varied between 30 and 120 °C (Fig. S2B, in Electronic Supplementary Information). In addition, Fig. S3 (in Electronic Supplementary Information) shows that there is no significant change in potential at a volume ratio of 6:1 to 8:1. For environmental friendliness and economy, AuNPs were prepared at 70 °C with a volume ratio of NMP*:HAuCl_4_ at 6:1. The absorption values (700 nm) at different times were measured under the condition that the Ionic strength was controlled below 20 mM and pH 7 to study the stability of AuNPs. As shown in Fig. S4 (in Electronic Supplementary Information), within 25 days, AuNPs remained stable and their absorption values changed weakly (increasing by 0.003), indicating that the AuNPs had good stability.

### Optimization of detection conditions

In order to realize efficient and sensitive detection of Hg^2+^, the effect of AuNPs concentration, pH and ionic strength on the experimental results were explored.

#### Effect of AuNPs concentration

Different concentration of AuNPs have different sensitivity to Hg^2+^ detection, the effect of AuNPs concentration on Hg^2+^ detection was first determined. The concentration of freshly prepared AuNPs stock solution was C_0_, and when the concentration of AuNPs fluctuated in the range of C_0_ to 1/6C_0,_ recorded the absorbance change value (ΔA) at 700 nm. As illustrated in Fig. S5A (in Electronic Supplementary Information) as can be seen that with the decrease of AuNPs concentration, ΔA first increases and then decreases, reaching a peak at 1/2C_0_. Therefore, 1/2C_0_ was chosen as the optimal concentration of AuNPs.

#### Effect of pH

The pH of the system plays a major role in the sensing process. As shown in Fig. S5B (in Electronic Supplementary Information), the effect on Hg^2+^ detection by AuNPs was studied, when the pH of the system was varied between 2.0 and 11.0. It can be seen that the response of AuNPs to Hg^2+^ was relatively stable when the pH value was in the 4.0 to 7.0 range. Therefore, adjusting the pH of AuNPs to 7.0 was the first choice for the detection system.

#### Effect of ionic strength

In addition, the ionic strength may lead to the aggregation of AuNPs, which in turn affects the stability of AuNPs, resulting in false positive signals. Therefore, NaCl was used to adjust the ionic strength of the system, and the effect of ionic strength on the absorbance of AuNPs was studied. Fig. S5C (in Electronic Supplementary Information) shows that the absorbance (λ = 700 nm) of AuNPs were basically unchanged when the NaCl concentration was between 0 and 20 mM. The absorbance increased with the increasing NaCl concentration, which suggested that high ionic strength reduced the stability of AuNPs. Therefore, it was concluded that by controlling the ionic strength below 20 mM, false positive signal could be avoided.

### Sensitivity

**Figure 4:**
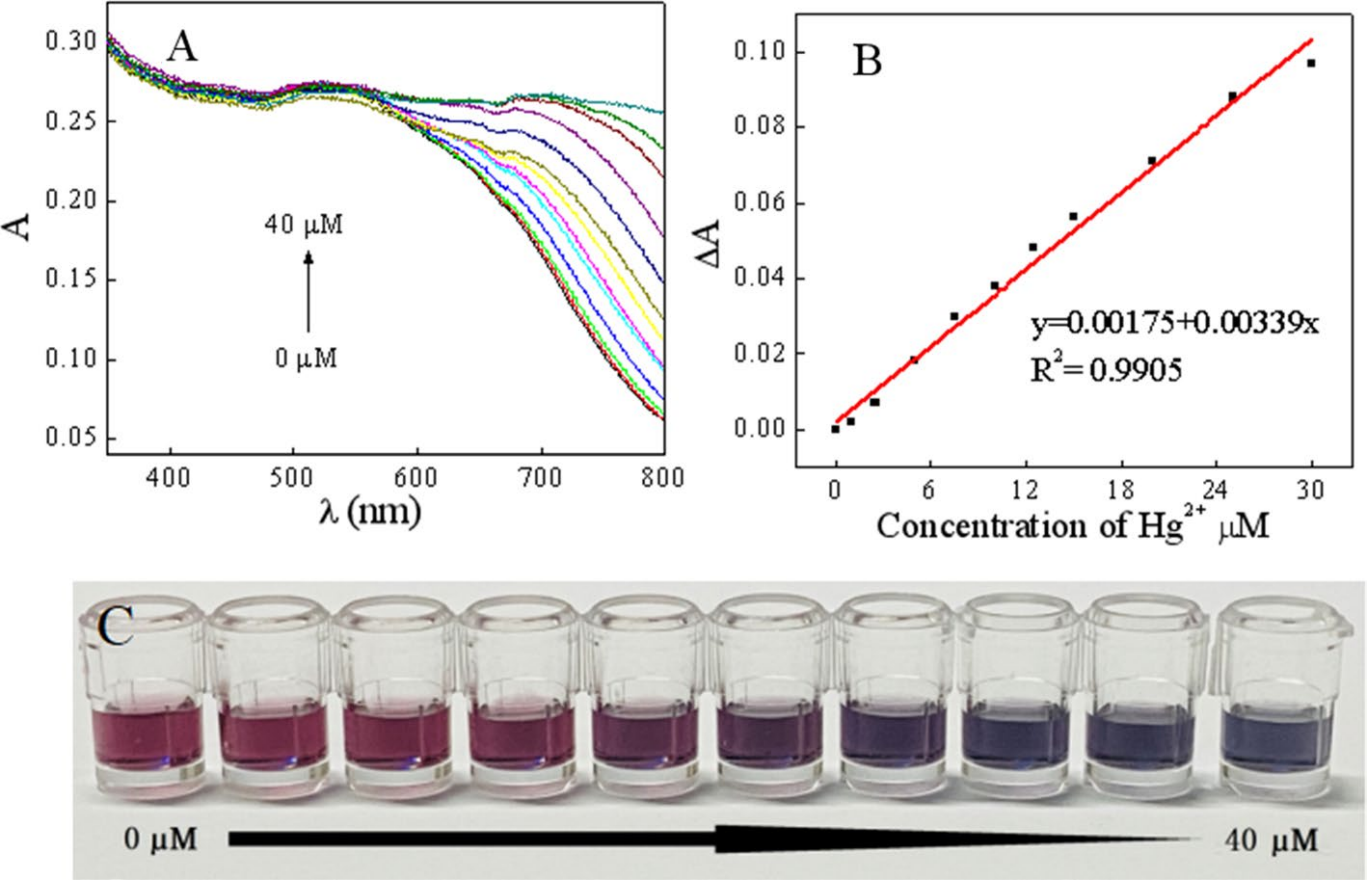
UV–Vis absorption spectra of AuNPs in the presence of Hg^2+^ with various concentrations (0, 1, 2.5, 5, 7.5, 10, 12.5, 15, 20, 25, 30, 35, 40 μM) (**A**), the linear response between absorbance change (ΔA) and Hg^2+^ concentration (**B**) and photographs of AuNPs in the presence of different concentrations of Hg^2+^ (**C**).

In order to explore the response of the colorimetric sensor to Hg^2+^ and LOD, the UV–Vis absorption spectra of AuNPs and different concentrations of Hg^2+^ were studied under optimal experimental conditions. From Fig. 4A, it can be found that the absorbance value at 700 nm gradually increased with the increase of the Hg^2+^ concentration, which was due to the fact that increase in the number of aggregated AuNPs with the increase of the Hg^2+^ content, thereby increasing the absorbance. Meanwhile, Fig. 4B described the linear response of Hg^2+^ concentrations at 1–30 μM, and the linear regression equation can be expressed as ΔA = 0.00175 + 0.00339 [Hg^2+^]. The LOD value for 0.3 µM (0.06 ppm) was determined from the formula: LOD = 3.29 S_B_/m, where S_B_ and m are the standard deviation of the blank and the slope of the calibration curve, respectively. As shown in Fig. 4C, these changes in the UV–vis spectra correspond to a gradual change in AuNPs color from wine-red to blue-gray, and the estimated visual LOD is 15 μM (3 ppm). Compared to other methods, this method has better sensitivity (Table S1, in Electronic Supplementary Information).

### Selectivity

**Figure 5:**
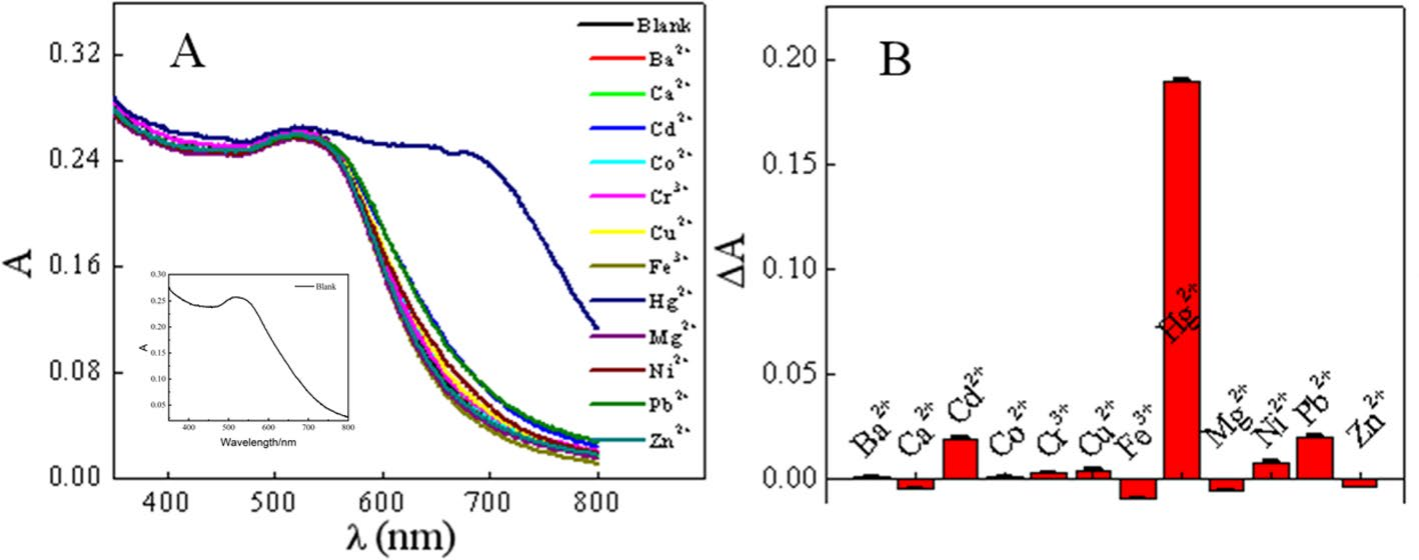
AuNPs selectivity study for Hg^2+^, UV–Vis absorption spectra (**A**) Histogram at 700 nm (**B**).

To assess the specificity of the proposed colorimetric assay for Hg^2+^ determination, the response to several potential coexisting interfering metal ions, inorganic species, and biomolecules was investigated without the addition of masking agents. From the UV–Vis absorption spectra in Fig. 5A and Fig. S6A (in Electronic Supplementary Information), it can be found that only Hg^2+^ significantly increases the absorbance of AuNPs at 700 nm. At the same time, Fig. 5B and Fig. S6B (in Electronic Supplementary Information) also show that the potential interfering substances have minimal impact on the detection of Hg^2+^, and thus the colorimetric sensor has good specificity for Hg^2+^. From this fact, it can be concluded that among these general metal ions, only Hg^2+^ can cause the aggregation of AuNPs, which provides the possibility for the efficient and selective for Hg^2+^ detection by colorimetry.

### Applications

Hg^2+^ is not only an environmental pollutant, but also can accumulate in the human body through the food chain, causing acute toxicity and damaging human health. Therefore, the detection of Hg^2+^ is of great importance to the environment and food safety. To test the potential application of AuNPs to detect Hg^2+^, three environmental water samples were tested using the standard spiking method. The results are shown in Table 1. The recoveries of Hg^2+^ in the spiked water samples ranged between 90.5 and 108.7%, and the relative standard deviations ranged between 0.78 and 1.87%, indicating that the colorimetric sensor has great potential in determining the Hg^2+^ concentrations in relation to the environment.

**Table 1:** Determination of Hg^2+^ in environmental water samples.

Sample	Present method			
	Added μM	Found μM	Recovery %	RSD %
Tap water	0.0	–	–	–
	10.0	9.1	90.5	1.87
	20.0	21.7	108.2	0.78
Lake water	0.0	–	–	–
	10.0	9.4	97.3	1.81
	20.0	21.4	108.7	0.80
River water	0.0	–	–	–
	10.0	9.7	99.3	1.75
	20.0	20.9	105.7	1.63

## Possible mechanism

*N*-Methyl-2-pyrrolidone can be oxidized into peroxides in the presence of O_2_ and H_2_O, and these unstable oxides will be converted into stronger reducing agent 5-hydroxypyrrolidone. This secondary alcohol can be used as a reducing agent, then Chloroauric acid was reduced to form gold nanoparticles. In short, Lactam groups are oxidized in the presence of oxygen and at high temperatures, peroxides are formed and further oxidized to form secondary alcohols, which can be used as reducing agents. This is also the reason for choosing NMP^34,35^.

AuNPs continuously form Au-Hg in the presence of Hg^2+^, leading to the aggregation of AuNPs^36^, and increased absorption at 700 nm. Through zeta potential measurement, it was found that the potential of the synthesized AuNPs is − 17.03 mV, while Hg^2+^ is 2.44 mV, after the addition of Hg^2+^, the potential changes to − 16.27 mV. Fig. S7 (in Electronic Supplementary Information) shows that the particle size significantly increases after the addition of Hg^2+^ (Fig. S8, in Electronic Supplementary Information), combined with Fig. 3B, the appearance of large aggregates can confirm that the addition of Hg^2+^ triggers the aggregation of AuNPs.

## Conclusions

In this paper, a colorimetric probe AuNPs with direct response to Hg^2+^ was developed using treated having stronger reducibility NMP as reducing and stabilizing agent. The presence of Hg^2+^ can cause the aggregation of AuNPs and increase the absorbance at 700 nm. The absorbance value of AuNPs showed a good linear relationship with Hg^2+^ in the concentration range of 0–30 μM (R^2^ = 0.9905), the LOD at 0.3 μM. At the same time, the wine-red AuNPs turned to blue-gray, and the estimated visual LOD at 15 μM. The method has good specificity for Hg^2+^, which provides a firm foundation for the successful quantitative analysis of Hg^2+^ in environmental sewage samples.

### Supplementary Information

Supplementary Information.

## Data Availability

The data generated and analysed during this study are included in the published article and its supplementary information file. All files can be provided by corresponding author on reasonable request.
