# Supplementary material for: A colorimetric detection of Hg2+ based on gold nanoparticles synthesized oxidized N-methylpyrrolidone as a reducing agent
Source: Sci Rep. 2023 Dec 14;13:22208. doi: 10.1038/s41598-023-49551-x (PMC10721636; doi:10.1038/s41598-023-49551-x)
Supplement: Supplementary file 1 — Supplementary Information. [file 41598_2023_49551_MOESM1_ESM.doc]

A colorimetric detection of Hg^2+^ based on gold nanoparticles synthesized oxidized N-methylpyrrolidone as a reducing agent

Xiaodong Shao^1,2^, Dou Yang^3^, Min Wang^3^, Qiaoli Yue^3^*

^1^State Key Laboratory of Performance and Structural Safety for Petroleum Tubular Goods and Equipment Materials, CNPC Tubular Goods Research Institute, Xi’an 710077, China

^2^School of Chemistry and Chemical Engineering, Northwestern Polytechnical University, Xi’an 710129, China

^3^School of Chemistry and Chemical Engineering, Shandong Provincial Key Laboratory of Chemical Energy Storage and Novel Cell Technology, Liaocheng University, Liaocheng 252059, China

Corresponding author:

Dr. Qiaoli Yue

Email, yueqiaoli@lcu.edu.cn

Fig. S1 FT-IR spectroscopy (A) and HR-XPS pattern (B) of AuNCs

Fig. S2 Effect of synthesis conditions on the absorbance of AuNPs including precursor volume ratio (A) and reaction temperature (B)


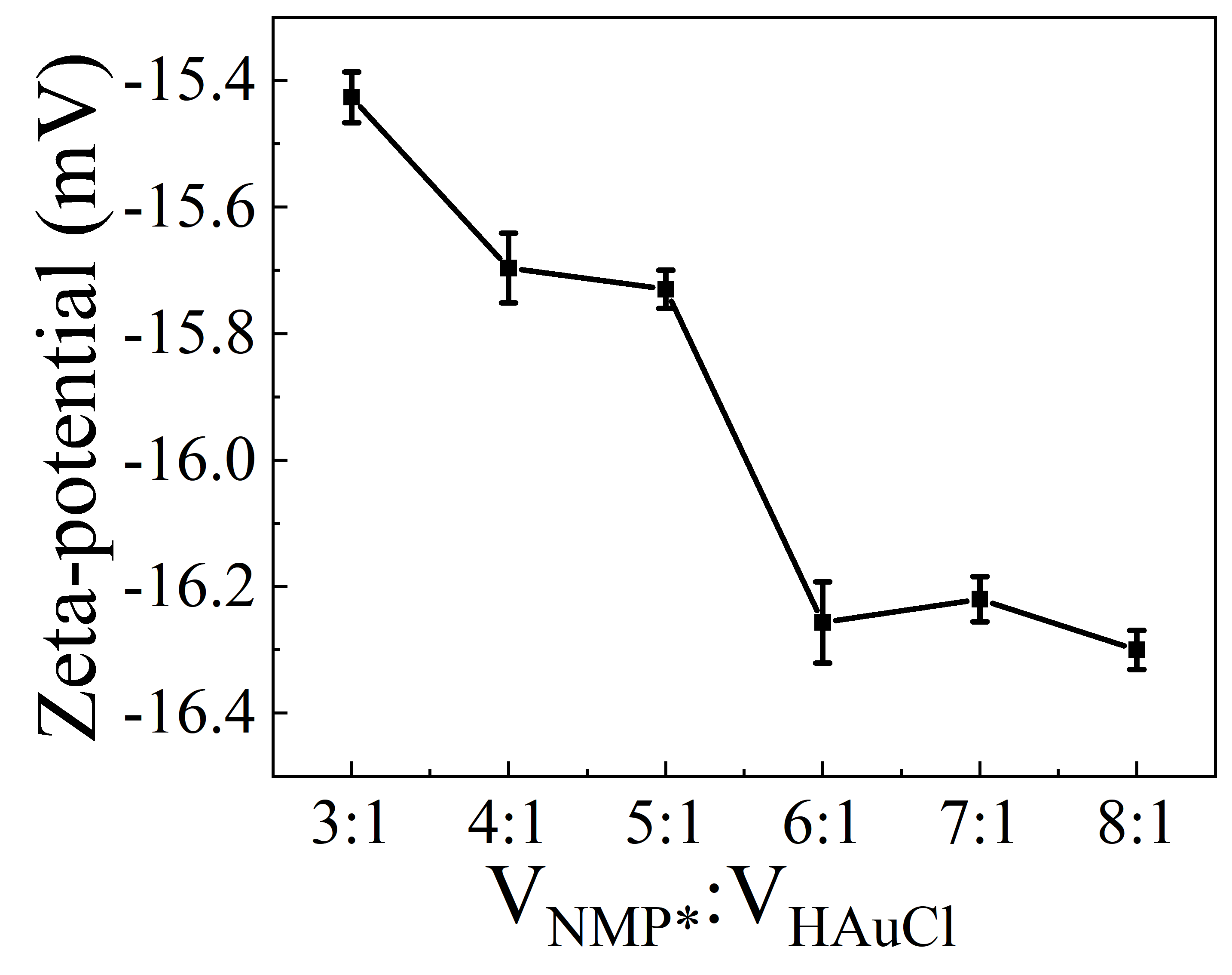


Fig. S3 Effect of precursor volume ratio on the Zeta potential of AuNPs


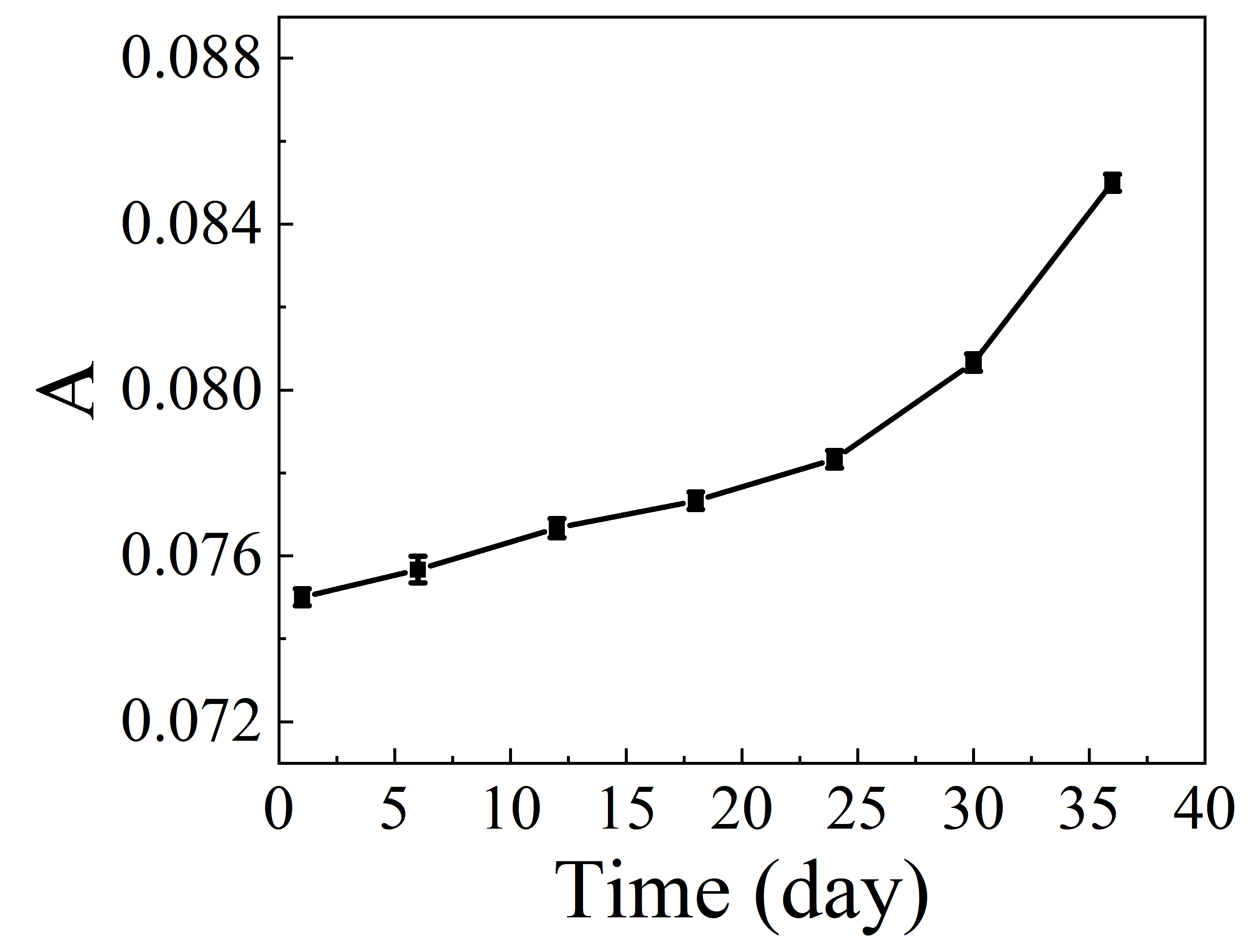


Fig. S4 Absorbance of AuNPs stored at different time

Fig. S5 Effect of AuNPs concentration (A), pH (B) and ionic strength (C) on the absorbance of AuNPs

Fig. S6 AuNPs selectivity study for Hg^2+^ (A) absorption value of different inorganic species, and biomolecules and. (B) antiinterference of NZC-CDs to detect
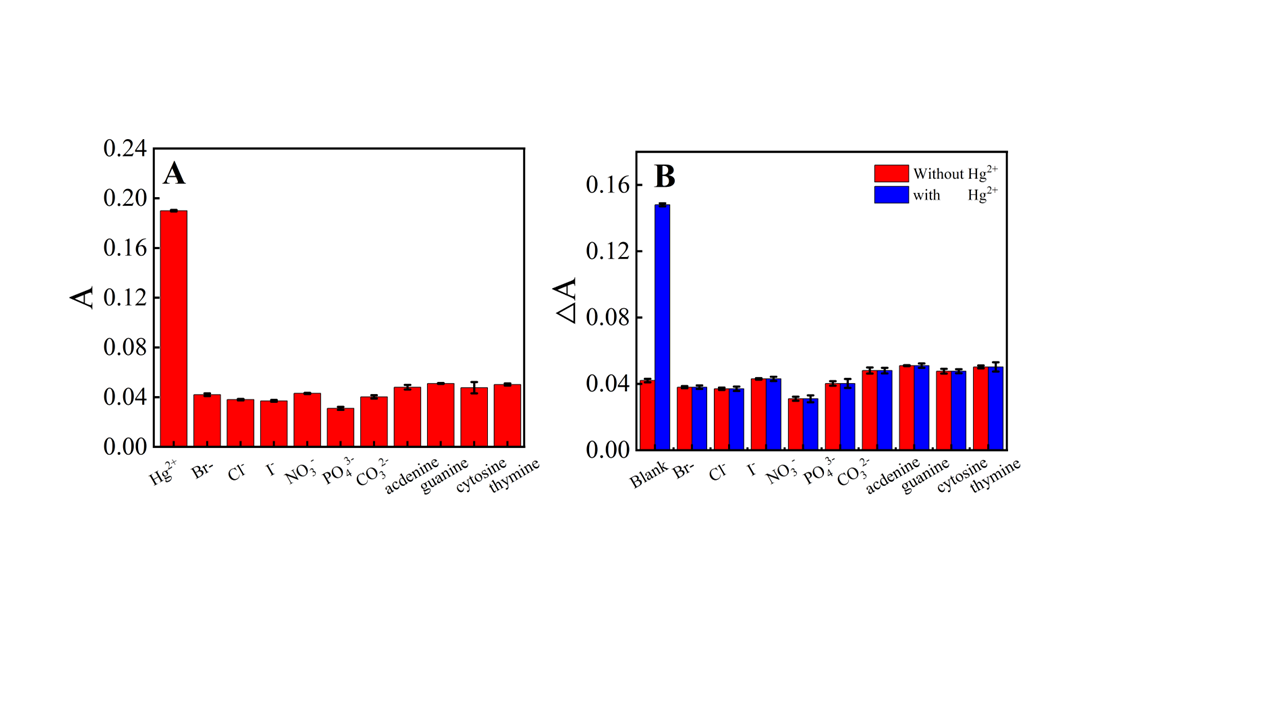
Hg^2+^


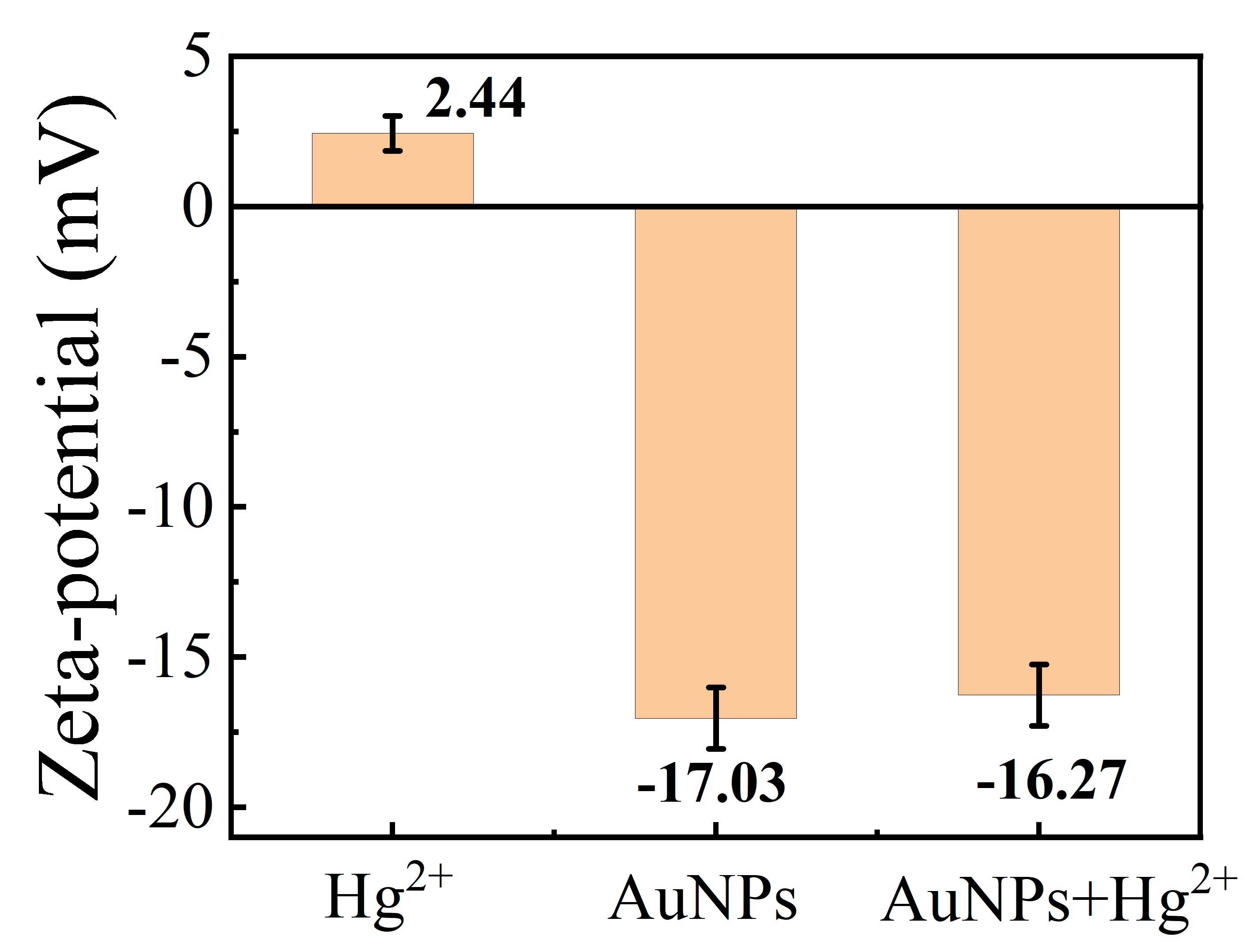


Fig. S7 Zeta-potential of Hg^2+^, AuNPs and AuNPs+Hg^2+^


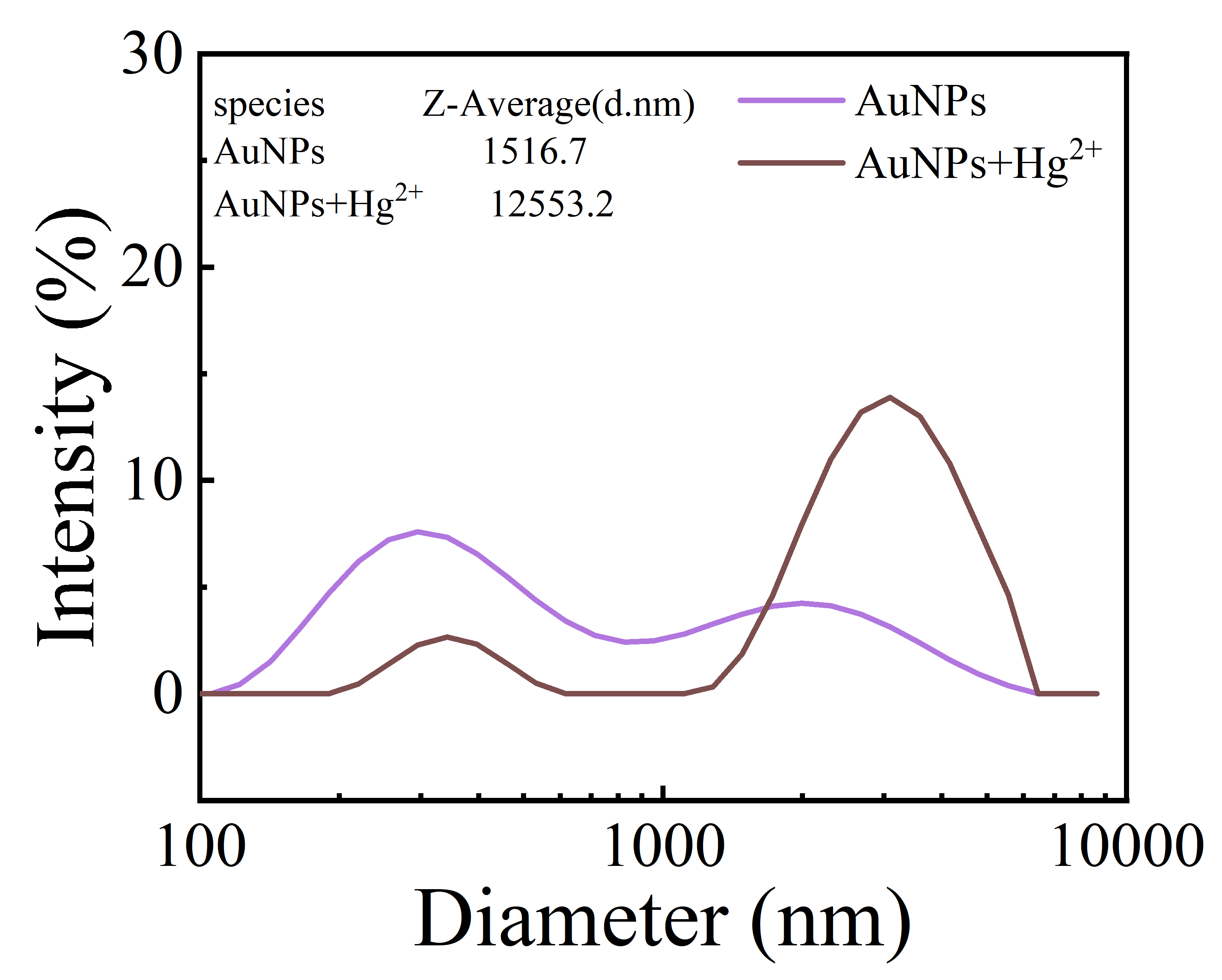


Fig. S8 Particle size distribution of AuNPs and AuNPs+Hg^2+^

| Table S1 Results for the detection of Hg^2+^ with different materials | | | |
| --- | --- | --- | --- |
| Materials | Linear range (μM) | LOD (μM) | Reference |
| FLN | 0-1.3 | 0.04 | [1] |
| Ru1 | 0-50 | 0.95 | [2] |
| thiophenol amine | 0.32-7.59 | 0.18 | [3] |
| Ru1 | 0-8 | 0.095 | [4] |
| C-Dots | 50-100 | 0.032 | [5] |
| AuNPs | 0-30 | 0.3 | This work |

**References**

1. L. Huang, Y. Sun, G. Zhao, L. Wang, X. Meng, J. Zhou, H. Duan, A novel fluorescein-based fluorescent probe for detection Hg^2+^ and bioimaging applications, Journal of Molecular Structure 1255 (2022) 132427.
2. C. Xu, K. Du, Y. Wu, L. Tan, X. Li, A cycloruthenated complex: Detecting Hg2+ by Hg2+‐promoted coordination switch and Cu2+ by coordination, Applied Organometallic Chemistry 36(9) (2022) e6806.
3. N. Sidana, P. Devi, H. Kaur, Thiophenol amine-based Schiff base for colorimetric detection of Cu^2+^ and Hg^2+^ ions, Optical Materials 124 (2022) 111985.
4. C. Xu, K. Du, Y. Wu, L. Tan, X. Li, A cycloruthenated complex: Detecting Hg^2+^ by Hg^2+^-promoted coordination switch and Cu^2+^ by coordination, Applied Organometallic Chemistry 36(9) (2022).
5. H. Zhang, S. Wu, Z. Xing, H.-B. Wang, Turning waste into treasure: chicken eggshell membrane derived fluorescent carbon nanodots for the rapid and sensitive detection of Hg^2+^ and glutathione, The Analyst 146(23) (2021) 7250-7256.
